# Circulating Exosomal microRNAs as Biomarkers of Systemic Lupus Erythematosus

**DOI:** 10.6061/clinics/2020/e1528

**Published:** 2020-08-21

**Authors:** Wengen Li, Sudong Liu, Yongyu Chen, Ruiqiang Weng, Ke Zhang, Xuechun He, Chunmei He

**Affiliations:** I Rheumatology Department, Meizhou People's Hospital (Huangtang Hospital), Meizhou Hospital Affiliated to Sun Yat-sen University, Meizhou 514031, P. R. China.; II Research Experimental Center, Meizhou People's Hospital (Huangtang Hospital), Meizhou Hospital Affiliated to Sun Yat-sen University, Meizhou 514031, P. R. China.; III Guangdong Provincial Key Laboratory for Precision Medicine and Translational Research of Hakka Population, Meizhou 514031, P. R. China.

**Keywords:** Systemic Lupus Erythematosus (SLE), Lupus Nephritis (LN), Exosomes, microRNAs, Biomarker

## Abstract

**OBJECTIVES:**

Many studies indicate that microRNAs (miRNAs) could be potential biomarkers for various diseases. The purpose of this study was to investigate the clinical value of serum exosomal miRNAs in systemic lupus erythematosus (SLE).

**METHODS:**

Serum exosomes were isolated from 38 patients with SLE and 18 healthy controls (HCs). The expression of miR-21, miR-146a and miR-155 within exosomes was examined by reverse transcription-quantitative polymerase chain reaction (RT-qPCR). Using receiver operating characteristic (ROC) curves, we evaluated the diagnostic value of exosomal miRNAs.

**RESULTS:**

Exosomal miR-21 and miR-155 were upregulated (*p*<0.01), whereas miR-146a expression (*p*<0.05) was downregulated in patients with SLE, compared to that in HCs. The expression of miR-21 (*p*<0.01) and miR-155 (*p*<0.05) was higher in SLE patients with lupus nephritis (LN) than in those without LN (non-LN). The analysis of ROC curves revealed that the expression of miR-21 and miR-155 showed a potential diagnostic value for LN. Furthermore, miR-21 (R=0.44, *p*<0.05) and miR-155 (R=0.33, *p*<0.05) were positively correlated with proteinuria. The expression of miR-21 was negatively associated with anti-SSA/Ro antibodies (R=−0.38, *p*<0.05), and that of miR-146a was negatively associated with anti-dsDNA antibodies (R=−0.39, *p*<0.05).

**CONCLUSIONS:**

These findings suggested that exosomal miR-21 and miR-155 expression levels may serve as potential biomarkers for the diagnosis of SLE and LN.

## INTRODUCTION

Systemic lupus erythematosus (SLE) is a chronic autoimmune disease characterized by abnormal autoantibody production and various clinical manifestations (1). Over the past decades, although the application of steroids and immunosuppressive drugs have largely improved SLE patient outcomes, the prevalence of SLE continues to increase annually. In addition, the severe organ-threatening manifestations of SLE, lupus nephritis (LN) and neuropsychiatric SLE (NPSLE), cause considerable harm to patients. Thus, early diagnosis of SLE is of great importance for preventing its progression.

Increasing evidence suggests that microRNAs (miRNAs) play crucial roles in the immune pathophysiology of SLE (2,3). miRNAs are a class of small non-coding RNAs with a length of 18 to 25 nucleotides; they regulate gene expression via multiple pathways (4). Abnormal expression of miRNAs contributes to immune disorders such as autoimmune and autoinflammatory diseases (5). Increasing numbers of circulating miRNAs have been confirmed as being dysregulated in SLE patients, and some of these miRNAs are related to clinical parameters (6,7). Recent studies show that circulating miR-21, miR-146a, and miR-155 participate in SLE progression and organ damage (7-9).

Exosomes are extracellular microvesicles with a diameter of 50-100 nm. Exosomes express specific molecules such as CD63 and CD81 on the surfaces of their membranes (8). Exosomes are secreted into various mammalian body fluids, such as blood, urine, saliva, and amniotic fluid (10). Previous studies have found that exosomes are rich in nucleic acids and proteins, which have made them potential biomarkers and drug delivery vectors for various diseases. Several studies have identified that exosomal miRNAs may also act as inter-cellular communication messengers (11,12). Wermuth et al. found that serum exosomes isolated from systemic sclerosis patients induced dermal fibroblasts to express pro-fibrotic genes and secrete fibronectin (13). Lin-Li et al. reported that the expression levels of exosomal miRNAs in the urine of patients with chronic kidney disease were significantly dysregulated, compared to those in the urine of healthy individuals (14). The levels of urinary exosomal miRNAs, which are found to be elevated in active LN patients, are considered as potential biomarkers of renal involvement in SLE (15). However, our understanding of the application of exosomal miRNAs as biomarkers for SLE remains poor.

In the present study, we analyzed the alterations of the expression levels of exosomal miR-21, miR-146, and miR-155 in SLE patients, compared to those in healthy controls (HCs). The purpose of this study was to identify potential novel biomarkers for SLE.

## MATERIALS AND METHODS

### Patients

Thirty-eight patients with SLE who attended the Rheumatology Department of the Meizhou’s People Hospital between 2018 and 2019 were recruited in our study. The patients were diagnosed with SLE according to the American College of Rheumatology classification criteria for SLE (16). Patients with chronic diseases that can cause kidney damage such as hypertension, diabetes, kidney stones, and hepatitis B were excluded. All patients with SLE enrolled in our study were diagnosed for the first time and did not receive medication. The SLE disease activity index (SLEDAI) was used to assess clinical disease activity (17), and LN was defined (proteinuria>0.5 g/day) according to the KDIGO Clinical Practice Guideline for Glomerulonephritis diagnostic criteria (18). Based on kidney involvement, the SLE patients were divided into two subgroups; LN and non-LN. The exclusion criteria were as follows: (i) complications involving malignant tumors and (ii) acute/chronic infections or other autoimmune diseases. The HCs were recruited from the Health Examination Center of the Meizhou People’s Hospital. The HCs did not exhibit any symptoms of SLE or renal damage, did not take any medication, and showed age and gender distributions similar to those of the SLE patients. This study was approved by the Ethics Committee of the Meizhou People’s Hospital. All participants signed written informed consent forms.

### Exosome isolation

Serum was isolated from fresh blood samples after centrifugation at 1,600 *g* for 10 minutes at 4°C and aliquoted into 1.5-ml sterile tubes. All serum specimens were stored at −80°C. Exosomes were extracted from serum using the ExoQuick kit (System Biosciences, CA, USA), following the manufacturer’s instructions. Briefly, 63 μl of ExoQuick solution was mixed with 250 μl of serum, followed by incubation at 4°C for 30 min, and then, centrifugation at 1,500 *g* for 30 min. The exosomes at the bottom were resuspended in 100 μl of phosphate-buffered saline. The exosomes were identified by western blotting using anti-CD63, anti-CD81, and anti-TSG101 antibodies (Abcam, UK), according to the manufacturer’s instructions.

### RNA extraction

Exosomal RNA was isolated using the Basic ExoRNA Kit (BioVision, USA), following the manufacturer’s instructions. The purity and concentration of total RNA was measured using a NanoDrop-1000 spectrophotometer (Thermo Fisher Scientific, Inc., Waltham, MA, USA). The exosomal miRNAs were stored at −80°C. In order to normalize possible variability, we spiked each denatured sample with 1 nmol of synthetic *C. elegans* miR-39 (*cel*-miR-39). The concentration and purity of the miRNA samples was assessed using the Qubit system (Thermo Fisher Scientific).

### Reverse transcription and miRNA quantification

Reverse transcriptase quantitative PCR (RT-qPCR) was performed using Bulge-LoopTM miRNA RT-qPCR Starter Kit (RiboBio Co., Ltd, Guangzhou, China) to measure the expression of the target miRNAs. Briefly, miRNA was polyadenylated and reverse-transcribed to cDNA. qPCR amplification was conducted in 96-well plates containing the cDNA products, SYBR Green master mix, and specific primers (RiboBio Co., Ltd, Guangzhou, China). The amplification reactions were carried out on a Roche LightCycler 480 platform (Roche, USA). The expression levels of the miRNAs were normalized to those of cel-miR-39 by the ΔΔCt method using the following formula: ΔΔCt=Ct[miRNA of interest] Ct[cel-miR-39]. The relative expression levels of miRNAs in patients were obtained following the 2-ΔΔCt method.

### Statistical analysis

SPSS 22.0 software was used for statistical analysis of the data. The data are presented as medians (25-75^th^ percentiles). Differences in clinical parameters between the groups were analyzed by Student’s t-test or Fisher’s exact test. The nonparametric Mann-Whitney U-test was used to analyze the expression of exosomal miRNAs between the SLE subgroups and HCs. Correlations between exosomal miRNAs and clinical parameters were assessed based on the Spearman’s rank correlation coefficient. Receiver operating characteristic (ROC) curves were generated to evaluate the diagnostic value of miRNAs with regard to SLE. *p*<0.05 was considered statistically significant.

## RESULTS

### Clinical characteristics of the study population

Thirty-eight SLE patients and 18 HCs participated in the present study. The age and gender of the subjects with LN, non-LN subjects, and HCs were not different. LN patients showed higher proteinuria levels and SLEDAI scores than non-LN patients. Compared to non-LN patients, both serum C3 and C4 levels at baseline were lower in LN patients; however, the differences were not significant. The demographic characteristics of the study subjects are shown in Table 1.

### Expression of exosomal miR-21, miR-155, and miR-146a in SLE patients and HCs

**Figure 1:**
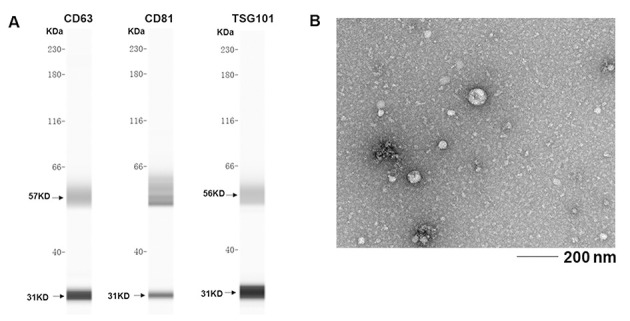
Western blotting analysis of the exosomal preparations showing the expression of the exosomal markers CD63, CD81, and TSG101.

To evaluate the role of exosomal miRNA expression in SLE patients, exosomes were extracted from serum and identified based on the presence of surface molecular markers. Exosomal markers, including CD63, CD81, and TSG101, were examined by western blotting (Figure 1A). The purified exosomes were further identified by electron microscopy (Figure 1B).

**Figure 2:**
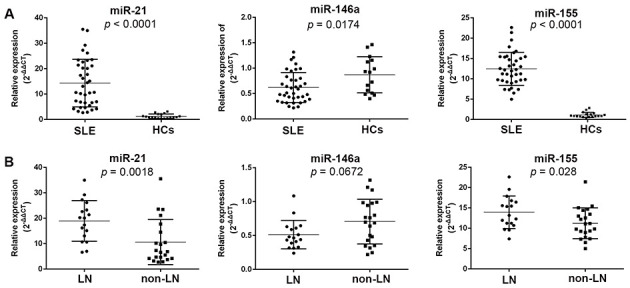
Relative expression of exosomal miRNAs in the study subjects. The relative expression levels of miR-21, miR-146a, and miR-155 are shown for SLE patients and HCs (A). Relative expression levels of miR-21, miR-146a, and miR-155 in the LN and non-LN subjects are shown (B). SLE: systemic lupus erythematosus; HCs: healthy controls; LN: lupus nephritis.

As shown in Figure 2, exosomal miR-21 and miR-155 were significantly elevated in SLE patients (*p*<0.0001 for both), whereas exosomal miR-146a levels were decreased in SLE patients (*p*=0.0174), compared to those in the HCs. Furthermore, we analyzed the exosomal miRNA expression levels among the subjects from the SLE subgroups. The LN patients exhibited higher levels of both miR-21 and miR-155 (*p*=0.0018 and 0.028, respectively).

### Diagnostic value of exosomal miR-21, miR-155, and miR-146a in SLE patients

**Figure 3:**
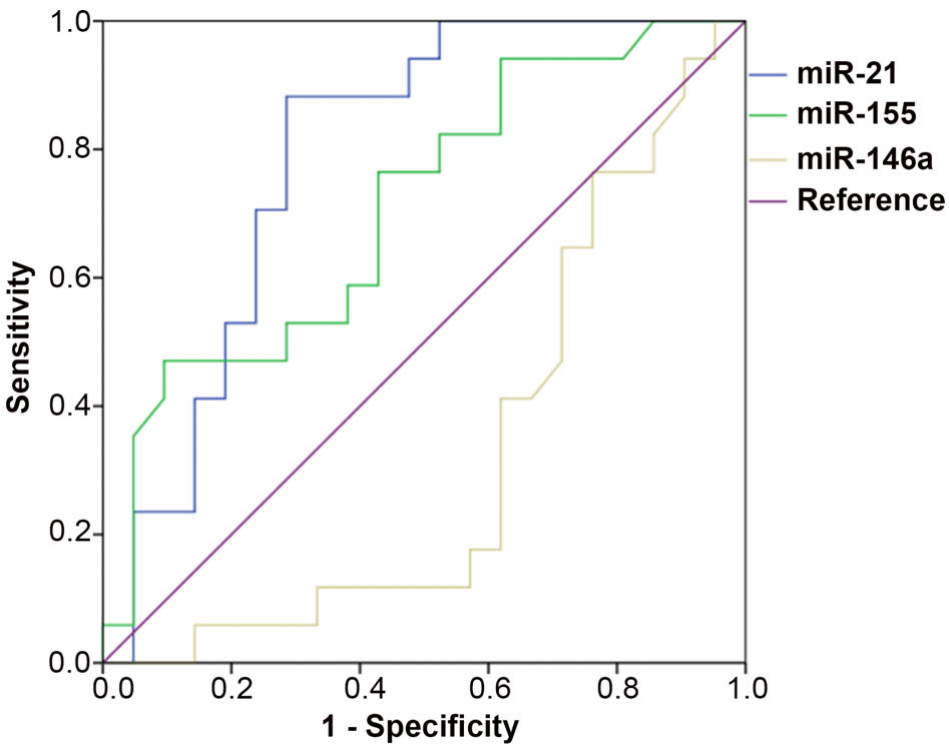
ROC curves were generated to analyze the sensitivity and specificity of exosomal miRNAs for distinguishing LN patients from non-LN patients. The AUC and *p* values of the miRNAs were as follows: miR-21 (AUC=0.790, *p=*0.002), miR-155 (AUC=0.709, *p=*0.029), and miR-146a (AUC=0.367, *p*=0.67). ROC: receiver operating characteristics; AUC: area under the curve; LN: lupus nephritis.

ROC curves were constructed to evaluate the diagnostic value of exosomal miRNAs in SLE patients. Significant differences were observed between SLE patients with LN and without LN, with an area under the curve (AUC) of 0.790 (95% CI: 0.643 to 0.937, *p*=0.002) for exosomal miR-21, and 0.709 (95% CI: 0.542 to 0.875, *p*=0.029) for exosomal miR-155. Differences in exosomal miR-146a expression between SLE patients with and without LN did not show statistical significance, with an AUC of 0.367 for miR-146a (95% CI: 0.150 to 0.500, *p*=0.067) (Figure 3). These results suggested that miR-21 and miR-155 may be used as potential biomarkers for the occurrence of LN in SLE patients.

### Correlation of exosomal miRNAs with clinical parameters

We further analyzed the correlations between serum exosomal miRNAs and various clinical parameters. Both exosomal miR-21 and miR-155 expression showed positive correlations with proteinuria (R=0.439, *p*=0.006 and R=0.330, *p*=0.043, respectively). Exosomal miR-21 was negatively correlated with the level of anti-SSA antibodies (R=−0.381, *p*=0.018). Exosomal miR-146a was negatively correlated with the erythrocyte sedimentation rate (ESR) (R=−0.370, *p*=0.022) and the level of anti-dsDNA antibodies (R=−0.391, *p*=0.015). Correlations between the expression of exosomal miRNAs and other clinical parameters were not statistically significant (Table 2).

## DISCUSSION

In the present study, we examined the expression of exosomal miR-21, miR-146a, and miR-155 in SLE patients. Our findings suggested that exosomal miR-21 and miR-155 are of clinical value in the diagnosis of SLE, both with and without LN. To our knowledge, studies concerning exosomal miRNAs being used as potential biomarkers in SLE are still limited.

It has been reported that miRNAs are very stable in blood circulation and are suitable as serum biomarkers for a variety of diseases (3). The sources and states of miRNAs in the circulation are heterogeneous. Many extracellular miRNAs have been revealed to be present in exosomes, which are microvesicles produced by many cells and can be detected in various body fluids (10). These exosomal miRNAs may serve as messengers between cells (12), and could thus, be used as circulating biomarkers for various human diseases (14). Dysregulation of miRNAs is found in several systemic autoimmune diseases, including SLE (9,19,20). Previous studies have suggested that exosomal miRNAs derived from SLE patients modulate inflammatory and adaptive immune responses (21).

MiR-155 has been widely studied in autoimmune diseases, but its functions remain controversial. Wang et al. found that miR-155 promotes autoimmune processes by inhibiting the expression of suppressor of cytokine signaling-1 (SOCS-1) (22). Aboelenein et al. reported that miR-155 inhibits autoimmunity by repressing transcription of *PU.1* and *TNF-α* (23). In our study, exosomal miR-155 expression was significantly upregulated in SLE patients, compared to that in HCs, and its expression was higher in LN patients than in non-LN patients. Meanwhile, exosomal miR-155 discriminated LN from non-LN patients, with an AUC of 0.709 (*p*=0.029). These results may imply that exosomal miR-155 contributes to the development of SLE. The exact mechanism underlying miR-155-mediated SLE development remains unclear. Jie et al. found that miR-155 inhibits the proliferation of mesangial cells and decreases the production of TGF-β1 in LN (24). Qian et al. reported that miR-155 deficiency ameliorates autoimmune inflammation in SLE by targeting sphingosine-1-phosphate receptor 1 (25).

miR-21 has been intensively studied in various autoimmune diseases, including SLE. Tang et al. observed that plasma miR-21 levels are elevated in newly diagnosed SLE patients, compared to those in healthy individuals (26). Another study conducted by Stagakis et al. found that active SLE patients show higher levels of miR-21 than inactive SLE patients, and miR-21 levels are correlated with SLEDAI scores. Further, miR-21 affects Programmed Cell Death 4 expression, and regulates aberrant T cell responses in human patients with SLE (27). A recent study revealed that exosomal miR-21 derived from SLE patients induces the production of type I interferons by human plasmacytoid dendritic cells (21). Our study found that the exosomal miR-21 expression was significantly upregulated in SLE patients, compared to that in the HCs, and that the expression of exosomal miR-21 was higher in LN patients than in non-LN patients. Exosomal mi-21 expression demonstrated a clinical value in discriminating LN patients from non-LN patients (AUC=0.790, *p=*0.002).

A number of studies (28,29) have suggested that miR-146a levels are decreased in SLE patients, while other studies have reported contrary findings (30,31). In the present study, we found that exosomal miR-146a expression was significantly downregulated in SLE patients, and was also negatively correlated with the anti-dsDNA antibody levels and the ESR.

The present study has a few limitations. First, the sample size in our study was not very large. Second, the present study identified the dysregulation of the expression of exosomal miR-21, miR-155, and miR-146a in SLE patients, but did not explore the mechanisms underlying this dysregulation. We will focus on such mechanisms in our future research.

## CONCLUSION

In conclusion, the present study analyzed the expression levels of circulating exosomal miR-21, miR-146a, and miR-155 in SLE patients. Our data suggested that exosomal miR-21 and miR-155 may serve as potential biomarkers of SLE with and without LN.

## AUTHOR CONTRIBUTIONS

Li W and Liu S designed the project and analyzed the data. Li W wrote the manuscript drafting. Liu S reviewed and corrected the manuscript. Chen Y and Weng R conducted experiments and contributed to the data collection. Zhang K, He X and He C contributed to enrolling patients, and collecting blood samples and clinical data.

## Figures and Tables

**Table 1:** Demographic and clinical parameters of systemic lupus erythematosus (SLE) patients and healthy controls (HCs).

Variables	SLE (n=38)		HCs (n=18)
	LN (n=17)	Non-LN (n=21)	
Age (years) (mean±SD)	33 (24-40)	30 (25-38)	30.5 (23.8-34)
Gender: male/female	1/16	2/19	2/16
White blood cells (×1000)	3.9 (2.5-5.4)	4.6 (3.3-7.2)	n/a
Hemoglobin (mg/dL)	9.9 (8.4-10.5)	9.7 (9.1-11.6)	n/a
Hematuria	11 (64.7%)	8 (38.1%)	n/a
Proteinuria level (g/24 h)	1.21 (0.71-2.03)	0.33 (0.23-0.41)**	n/a
Creatinine (mg/dL)	0.81 (0.71-0.88)	0.78 (0.72-0.89)	0.71 (0.68-0.74)
ESR (mm/h)	54 (26-89)	53 (18-84)	n/a
Low C3	17 (100.0%)	20 (95.2%)	n/a
Low C4	15 (88.2%)	15 (71.40%)	n/a
Anti-dsDNA Ab (+), n (%)	16 (94.1%)	18 (85.7%)	n/a
Anti-Sm Ab (+), n (%)	9 (52.9%)	15 (71.4%)	n/a
Anti-SSA/Ro Ab (+), n (%)	15 (88.2%)	16 (76.2%)	n/a
Anti-SSB/La Ab (+), n (%)	3 (17.6%)	6 (28.6%)	n/a
SLEDAI	19 (15-22)	10 (8-14)**	n/a

**p<0.01, comparison between LN and non-LN subjects.

n/a: not applicable;

ESR: erythrocyte sedimentation rate;

C3: complement component 3;

C4: complement component 4;

SLEDAI: systemic lupus erythematosus disease activity index.

**Table 2:** Spearman’s rank correlation coefficients between exosomal miRNA expression levels and various clinical parameters in SLE patients.

Variables	miR-21		miR-146a		miR-155	
	R	*p*	R	*p*	R	*p*
Creatinine	−0.182	0.274	−0.139	0.404	−0.072	0.668
ESR	0.037	0.826	−0.370	0.022*	−0.085	0.613
C3	0.015	0.931	0.219	0.186	0.036	0.823
C4	0.299	0.069	0.192	0.248	0.060	0.723
Proteinuria	0.439	0.006**	−0.220	0.183	0.330	0.043*
Anti-dsDNA Ab	−0.078	0.641	−0.391	0.015*	−0.070	0.675
Anti-RNP Ab	−0.115	0.490	0.010	0.954	−0.139	0.404
Anti-Sm Ab	−0.119	0.475	0.082	0.624	0.075	0.656
Anti-SSA Ab	−0.381	0.018*	0.040	0.810	0.170	0.307
Anti-SSB Ab	−0.260	0.115	−0.093	0.578	−0.124	0.458
SLEDAI	−0.063	0.709	−0.238	0.151	−0.075	0.654

*p<0.05, **p<0.01, comparison by Student’s t-test, R=correlation coefficient.

ESR: erythrocyte sedimentation rate;

C3: complement component 3;

C4: complement component 4;

SLEDAI: systemic lupus erythematosus disease activity index.
